# Investigating institutional abuse survivors’ help-seeking attitudes with the Inventory of Attitudes towards Seeking Mental Health Services

**DOI:** 10.1080/20008198.2017.1377528

**Published:** 2017-10-12

**Authors:** Viktoria Kantor, Matthias Knefel, Brigitte Lueger-Schuster

**Affiliations:** ^a^ Faculty of Psychology, University of Vienna, Vienna, Austria

**Keywords:** Trauma survivors, institutional abuse, attitudes, PTSD, mental health service use, sobrevivientes de trauma, abuso institucional, actitudes, 24 TEPT, uso de servicios de salud mental, 创伤幸存者, 机构内虐待, 态度, 24 PTSD, 精神健康服务使用, • The three-factor structure of IASMHS was supported by a confirmatory factor analysis.• Institutional abuse survivors’ current mental health service use was predicted by the help-seeking propensity scale of IASMHS, higher levels of PTSD intrusion, and lower levels of depression.• Early post-trauma interventions should target trauma survivors’ help-seeking propensity to facilitate mental health service use.

## Abstract

**Background**: Although effective treatments exist, many trauma survivors delay or avoid professional help. Attitudes towards help-seeking are associated with intentions to and actual treatment use, but were neglected in research on trauma survivors so far.

**Objective**: This study aimed to investigate the reliability, construct validity, and predictive power of the Inventory of Attitudes towards Seeking Mental Health Services (IASMHS) and to investigate attitudes of adult institutional abuse survivors.

**Method**: A total of 220 adult survivors of institutional abuse were interviewed using IASMHS, the PTSD Checklist for DSM-5 (PCL-5), the Childhood Trauma Questionnaire Short-Form (CTQ-SF), the Life Events Checklist (LEC-5), and the depression-subscale of the Brief Symptom Inventory (BSI-18). They were further asked about their current mental health service use. We assessed the fit of different models of IASMHS with confirmatory factor analyses and predicted current mental health service use with a binominal logistic regression model.

**Results**: The three-factor structure of IASMHS provided the best fit. One of the three scales (help-seeking propensity), the PTSD-intrusion scale, and the depression scale significantly contributed to the prediction of current mental health service use. Single items of the psychological openness scale loaded weakly on the according factor. Our sample showed a similar IASMHS profile compared to other samples with mental health problems.

**Conclusion**: Overall, IASMHS appears to be a useful instrument to assess attitudes towards seeking mental health services in trauma survivors. It can be used to investigate help-seeking attitudes and its correlates to better understand and facilitate survivors’ treatment use.

## Abstract (es)

**Planteamiento**: Aunque existen tratamientos eficaces, muchos sobrevivientes de trauma retrasan o evitan ayuda profesional. Las actitudes respecto a buscar ayuda están asociadas con las intenciones y el uso real del tratamiento, pero, hasta el momento, se han descuidado en la investigación con muestras de trauma.

**Objetivo**: El presente estudio tuvo como objetivo investigar la fiabilidad, la validez de constructo y el poder predictivo del Inventario de actitudes respecto a búsqueda de servicios de salud mental (IASMHS, siglas de *Inventory of Attitudes towards Seeking Mental Health Services*) e investigar las actitudes de los adultos que han sobrevivido al abuso institucional.

**Método**: Un total de 220 adultos sobrevivientes de abuso institucional fueron entrevistados usando el IASMHS, la Lista de Verificación del TEPT para el DSM-5 (PCL-5, siglas de *PTSD Checklist for DSM-5*), la versión corta del Cuestionario sobre trauma infantil (CTQ-SF, siglas de *Childhood Trauma Questionnaire Short-Form*), la Lista de verificación de acontecimientos vitales (LEC-5; siglas de *Life Events Checklist*) y la sub-escala de depresión del Inventario breve de síntomas (BSI-18, siglas de *Brief Symptom Inventory*). También se les preguntó por su uso actual de servicios de salud mental. Se evaluó cómo encajaban los diferentes modelos del IASMHS con análisis de factores de confirmación y se predijo el uso actual de servicios de salud mental con un modelo de regresión logística binominal.

**Resultados**: La estructura de tres factores del IASMHS fue la que mejor encajó. Una de las tres escalas (propensión a la búsqueda de ayuda), la escala de intrusión del TEPT y la escala de depresión contribuyeron significativamente a la predicción del uso actual de servicios de salud mental. Los elementos individuales de la escala de apertura psicológica se cargaron débilmente en el factor correspondiente. Nuestra muestra mostró un perfil similar del IASMHS en comparación con otras muestras con problemas de salud mental.

**Conclusión**: En general, el IASMHS parece ser un instrumento útil para evaluar las actitudes respecto a la búsqueda de servicios de salud mental en los sobrevivientes de trauma. Puede utilizarse para investigar las actitudes de búsqueda de ayuda y sus correlatos para comprender mejor y facilitar el uso del tratamiento a los supervivientes.

## Abstract (zh)

**背景：**尽管有效的治疗存在，许多创伤幸存者延迟或者回避专业帮助。求助态度和对治疗的使用意图以及实际使用有关，但在创伤样本的研究中至今被忽视。

**目标：**本研究旨在考察使用精神健康服务的求助态度清单（IASMHS）的信效度和预测能力，以及机构内虐待的成年幸存者的求助态度。

**方法：**使用IASMHS、DSM-5 PTSD检查表（PCL-5）、短版童年创伤问卷（CTQ-SF）、生活事件检查表（LEC-5）和简明症状清单（BSI-18）的抑郁分量表对总共220名机构内虐待的成年幸存者进行访谈。更一步询问了他们目前的精神健康服务的使用。我们使用验证性因素分析评估了IASMHS的不同模型，并使用二项式Logistic回归分析考察其对目前精神健康服务使用的预测能力。

**结果：**IASMHS的三因子结构拟合最佳。其中的求助倾向量表（help-seeking propensity）、PTSD的闯入量表和抑郁量表对预测目前精神健康服务使用情况有显著贡献。心理开放（psychological openness）题目在相应的因子上载荷较低。我们的样本和其它具有精神健康问题的样本一样，显示相似的IASMHS剖面结构。

**结论：**总体上，IASMHS是评估创伤幸存者使用精神健康服务求助态度的有用工具。它可以用来考察求助态度，有助于更好理解和促进幸存者的治疗使用行为。

## 1. Introduction

Being exposed to or witnessing traumatic events such as natural or technical disasters, abuse, or violent attacks can lead to posttraumatic stress disorder (PTSD) and can increase the risk for other mental disorders (Perkonigg, Kessler, Storz, & Wittchen, 2000). Moreover, untreated trauma related disorders can be persistent and may have adverse consequences on trauma survivors’ individual lives, and on society according to socioeconomic costs. Although various effective trauma treatments exist (Kitchiner, Roberts, Wilcox, & Bisson, 2012; Van Etten & Taylor, 1998; Watts et al., 2013), the majority of individuals with trauma related mental disorders do not receive professional help (Kessler, 2000). This concerning mental health care situation requires a better understanding of the factors that are involved in mental health service use of trauma survivors. Mental health services typically comprise professional help from mental health specialists such as psychiatrists, psychologists, or psychotherapists (Kessler et al., 2005; Mackenzie, Erickson, Deane, & Wright, 2014).

Three key factors were identified to be strongly associated with mental health service use after traumatic events: higher levels of psychopathology, type and degree of the traumatic event, and female gender (Gavrilovic, Schützwohl, Fazel, & Priebe, 2005). To address the lack of knowledge towards reasons for mental health service use, Gavrilovic et al. (2005) emphasized a closer investigation of trauma survivors’ attitudes towards services to better understand their mediating and moderating effects on seeking professional help. In general, positive and negative attitudes towards seeking mental health services influence intentions to seek professional help and actual health care use (Freitas-Murrell & Swift, 2015). A cross-temporal meta-analysis (1968–2008) showed that help-seeking attitudes changed in a disconcerting direction and became increasingly negative during the investigated period (Mackenzie et al., 2014). In a study on attitudes towards mental health service use in six European countries, ten Have et al. (2010) reported that one-third of participants believed professional care for mental health problems was equitable to no help or even worse. These findings show that more effort is needed to investigate attitudes towards mental health service use, with the goal to better understand, predict, and facilitate individuals’ help-seeking for professional care.

### 1.1. Attitudes towards seeking mental health services

Fishbein and Ajzen (1975) described that an individuals’ intention to perform a certain behaviour is influenced by attitudes towards the behaviour and subjective norms (Theory of Reasoned Action). Their theory was later adapted in Ajzen’s (1985) Theory of Planned Behaviour, according to the role of perceived behavioural control, as a further factor that influences intentions. Following the Theory of Planned Behaviour, Mackenzie, Knox, Gekoski, and Macaulay (2004) adapted Fischer and Turner`s (1970) Attitudes Toward Seeking Professional Psychological Help Scale by extending the measure with items regarding the knowledge of subjective norms and perceived behavioural control towards seeking professional help. The resulting Inventory of Attitudes towards Mental Health Services (IASMHS) is a 24-item scale which is increasingly used in research.

Prior studies demonstrated various interactions between attitudinal factors and demographic characteristics: being female (e.g. Mackenzie, Gekoski, & Knox, 2006; ten Have et al., 2010) and being Caucasian (Ward, Wiltshire, Detry, & Brown, 2013) was correlated with more favourable attitudes. Young adults were reported to have less positive attitudes towards seeking professional help than older adults (Gonzalez, Alegria, & Prihoda, 2005; Robb, Haley, Becker, Polivka, & Chwa, 2003). Older adults have generally more positive attitudes, but they prefer physicians instead of specialized mental health services (James & Buttle, 2008; Mackenzie et al., 2006). A European epidemiological study of mental disorders reported that more positive attitudes towards mental health help-seeking were associated with being younger than 65 years (ten Have et al., 2010). This study did not find associations between educational levels and attitudes, while others did (Jagdeo, Cox, Stein, & Sareen, 2009; Riedel-Heller, Matschinger, & Angermeyer, 2005). Higher educated, older adults had more favourable attitudes regarding help-seeking propensity, but this positive effect of education on attitudes was only found for men (Mackenzie et al., 2006).

### 1.2. Inventory of Attitudes towards Seeking Mental Health Services (IASMHS; Mackenzie et al., 2004)

IASMHS comprises three scales: help-seeking propensity, psychological openness, and indifference to stigma. Help-seeking propensity refers to an individual’s intention and perceived ability to seek professional help for a psychological problem. Psychological openness represents an individual’s disposition to acknowledge psychological problems and to consider seeking professional help. The extent to which individuals are concerned about significant others opinion if they find out that they were receiving professional help is represented through the indifference to stigma subscale (Mackenzie, Knox, Gekoski, & Macaulay, 2004). Mackenzie et al. (2004) demonstrated that IASHMS discriminates between participants who had and those who had not used mental health services.

IASMHS has been used to investigate attitudes in college students (Mackenzie et al., 2004; Mojaverian, Hashimoto, & Kim, 2012; Wahto & Swift, 2016; Yousaf, Popat, & Hunter, 2015), adolescents (Munson, Floersch, & Townsend, 2010), older adults (Kessler, Agines, & Bowen, 2014), psychotherapy clients (Elkins, Swift, & Campbell, 2016), and specific ethnic groups: Alaska natives (Freitas-Murrell & Swift, 2015), South Asians (Loya, Reddy, & Hinshaw, 2010), Chinese immigrants (Tieu & Konnert, 2014), and African Americans (Ward et al., 2013; Watson & Hunter, 2015). Only a few studies screened for mental disorders (Mackenzie et al., 2006; Mesidor & Sly, 2014; Munson et al., 2010; Troeung, Gasson, & Egan, 2015; Ward et al., 2013; Watson & Hunter, 2015) and intentions to (or actual) mental health service use (Freitas-Murrell & Swift, 2015; James & Buttle, 2008; Martin & Howe, 2016; Tieu & Konnert, 2014). Two studies considered both mental disorders and intentions to/or actual mental health service use (Mackenzie et al., 2006; Mesidor & Sly, 2014). Three studies investigated trauma related samples: suicide bereaved adults (Drapeau, Cerel, & Moore, 2016), police officers (Hyland et al., 2014), and homeless and at-risk housed youth (Martin & Howe, 2016); none assessed trauma related disorders. Although some studies have been carried out on mental health service use of people with mental disorders, very little is known about trauma survivors’ attitudes towards service use. Since many trauma survivors with potentially treatable disorders seem to be reluctant to seek mental health services, we intended to investigate attitudes towards service use in a sample of adult survivors of institutional abuse with IASMHS. Institutional abuse survivors experienced sexual, physical, and/or emotional abuse in their early lives and many are affected by mental disorders and psychosocial problems in their adulthood (Fitzpatrick et al., 2010; Lueger-Schuster et al., 2013). As research on attitudes towards mental health service use in clinical samples is rather sparse, we intended to provide a first exploratory comparison of IASMHS scales in our trial with other psychological distressed samples.

The main aims of the present study were (1) to investigate the reliability and construct validity of IASHMS, (2) to investigate the power of IASMHS in predicting current mental health service use when also considering the effects of PTSD-symptoms, depression, age, and gender in the model, and (3) to discuss attitudes towards seeking professional help of institutional abuse survivors against the background of other samples with possible mental health problems.

## 2. Method

### 2.1. Background and procedure

Study participants were adult Austrian survivors of institutional abuse perpetrated during their childhood in Viennese foster care institutions. Institutional abuse is characterized by an omnipresent authority that exaggeratedly controls every aspect of children’s lives in institutionalized foster care settings (Goffman, 1987) and an inappropriate and potentially harmful use of power that threatens children`s well-being (Wolfe, Jaffe, Jette, & Poisson, 2003). The Austrian institutional abuse survivors reported experiences of abuse to a victim protection commission and were offered financial compensation and/or psychotherapy from the local government. In a next step, they were invited to participate in the larger Vienna Institutional Abuse Study (VIAS), which was designated to investigate long-term correlates of institutional abuse. Data for the present study came from VIAS. The study received ethical approval from the Ethics Committee of the University of Vienna and all participants gave written informed consent. Structured clinical interviews and questionnaires were administered by trained clinical psychologists in face-to-face interviews.

### 2.2. Participants

The sample of the present study consisted of 220 adult survivors of institutional abuse aged 29–87 years (*M* = 57.9, *SD* = 9.55; Q1 = 52, Q3 = 65; 40.0% women) and was compared with the Austrian population aged 55–64 years (Statistik Austria, 2016a, 2016b, 2016c) in order to contrast the sample characteristics with the average Austrian population structure. The majority of the sample was currently living alone (single = 15.0% [Austrian population: 9.8%], divorced = 35.0% [Austrian population: 15.2%], widowed = 5.9% [Austrian population: 6.0%]), while 44.1% (Austrian population: 69.2%) were living with a partner.

Compared with the Austrian population the educational levels were low: the majority of the sample (63.2%) completed an apprenticeship or a vocational school without an A-level degree (10–12 years of education without general qualification for university entrance; Austrian population: 49.8%), 19.5% attended compulsory school (nine years of education; Austrian population: 19.1%), 4.1% received an A-level education (12 years of education with general qualification for university entrance; Austrian population: 14.8%), 3.2% had a university degree (>15 years of education; Austrian population: 16.2%), and 10.0% did not complete compulsory school (<9 years of education; Austrian population: <1%).

Most of the study participants indicated that they were currently not employed: 26.8% were retired, 26.8% were early retired due to sickness or impairment (retired in total: 53.6%; Austrian population: 41.1%), 16.0% were unemployed and/or received social welfare benefits (Austrian population: 4.8%), 6.4% were in long-term sick-leave (Austrian population: 2.7%), and 3.2% were imprisoned (Austrian population: <0.01%). Overall 21.0% of the sample were currently employed (Austrian population: 44.5.3%).

All participants in the present study experienced at least one childhood trauma or potentially traumatic life event.

### 2.3. Measures

#### 2.3.1. Attitudes towards mental health service use

When interviewed with IASMHS (Mackenzie et al., 2004) participants are invited to rate their agreement with 24 statements on a five-point scale from ‘disagree’ (0) to ‘agree’ (4). For the current study we used the German version (Kessler et al., 2014). Kessler et al. (2014) preferred the term ‘professional psychological help’ to receiving mental health support from psychologists or psychiatrists. IASMHS was tested in a convenience sample of adults and showed good internal consistence (Cronbach’s *α* = .82) with α ranging from .76 to .82 in the three subscales. The test-retest reliability was investigated in a sample of undergraduate students and resulted in a total score of *r* = .85 (*p* < .01). The test-retest reliability for the single scales were as follows: psychological openness, *r* = .86, *p* < .01; help-seeking propensity, *r* = .64, *p* < .01; indifference to stigma, *r* = .91, *p* < .01 (Mackenzie et al., 2004). In the current study the internal consistence was: psychological openness, *α* = .67; help-seeking propensity, *α* = .80; indifference to stigma, *α* = .77; total score, *α* = .84.

#### 2.3.2. PTSD symptoms

The PTSD Checklist for DSM-5 (PCL-5; Weathers et al., 2013; Wortmann et al., 2016) assesses 20 PTSD symptoms on a five-point scale ranging from ‘not at all’ (0) to ‘extremely’ (5). PTSD is diagnosed if participants scored ‘moderately’ (2) or above on at least one re-experiencing symptom (B criterion), one avoiding symptom (C criterion), two negative alterations in cognitions and mood symptoms (D criterion), and two hyperarousal symptoms (E criterion).

The Childhood Trauma Questionnaire Short-Form (CTQ-SF; Bernstein et al., 2003) and the Life Events Checklist (LEC-5; Weathers et al., 2013) were used to assess the stressor-criterion (criterion A) of PTSD. In the CTQ-SF participants were asked to indicate, on a five-point scale ranging from ‘never true’ (1) to ‘very often true’ (5), if they were exposed to various childhood traumata (28 items); in the LEC-5 participants were asked if they experienced or witnessed potentially traumatic life events (17 items).

In the current study the psychometric properties were: Cronbach’s *α* = .79–.91 for the four PCL-5 subscales, Cronbach’s *α* = .94 for the total PCL-5 score, and Cronbach’s *α* = .93 for the total CTQ scale.

#### 2.3.3. Depression symptoms

We used the depression-subscale of the Brief Symptom Inventory (BSI-18; Derogatis, 2001), which was demonstrated to be an appropriate short-form of the original 53-item version (Derogatis & Melisaratos, 1983). Participants were asked to self-rate different symptoms they experienced over the last seven days on a five-point scale ranging from ‘not at all’ (0) to ‘extremely’ (4). The psychometric properties for the present study were good (Cronbach’s *α* = .88).

#### 2.3.4. Mental health service use

Current mental health service use was queried with the following question and a dichotomous response format: ‘Do you currently receive psychological treatment or psychotherapy?’ If participants answered ‘yes’ they were asked about the duration of the treatment in an open response format. If participants attended a minimum of at least eight sessions within 12 months we defined this as ‘receiving treatment’ (Wang, Demler, & Kessler, 2000; Wang et al., 2005).

### 2.4. Statistical analysis

Analyses were conducted with SPSS 22 (IBM Corp., 2013) and R-package lavaan (Rosseel, 2012) in statistical environment R (R Development Core Team, 2016). In a first step, we evaluated the psychometric properties of IASMHS by calculating the reliability index Cronbach’s α and by confirmatory factor analysis (CFA) to assess the fit of the data to different structural models of IASMHS. We estimated two CFA models (one- and three-factor CFA model, reflecting a general factor model and the three dimensions of IASMHS, respectively) and one bifactor model. Confirmatory bifactor modelling is a technique in which the item covariance is explained by a single general factor and two or more orthogonal ‘group’ factors (Reise, Moore, & Haviland, 2010). This model assumes that all items measure a common latent trait and thus all items load on one factor (attitudes towards seeking MHS); it furthermore assumes that the variance of all items is also influenced by independent factors reflecting the unique covariance among subsets of items (psychological openness, indifference to stigma, help-seeking propensity). This procedure was also applied by Hyland et al. (2014) and thus allows for a close comparison of the results.

In all models we used robust parameter estimation (weighted least square mean- and variance-adjusted estimator, WLSMV) to account for the ordered categorical structure of the item ratings and to provide robust parameter estimation, standard errors, and tests of model fit (Beauducel & Herzberg, 2006). Model fit was assessed using widely-used benchmarks (Hu & Bentler, 1999), utilizing the comparative fit index (CFI), the Tucker-Lewis index (TLI; CFI and TLI: good fit: ≥ .95, acceptable fit: ≥ .90), and the root mean square error of approximation (RMSEA; good fit: < .06, acceptable fit: < .08). To obtain the Bayesian Information Criterion (BIC) for comparison of the three different models, we reran the analyses with robust maximum likelihood estimation (MLR).

In a second step, we used binominal logistic regression analysis to predict current psychotherapy use. We used the three scales of IASMHS (psychological openness, help-seeking propensity, indifference to stigma) as predictors in the regression model. To consider the previously reported associations of psychopathological symptom burden, age, and gender, we also included the four PTSD dimensions (intrusions, avoidance, negative alterations in cognitions and mood, alterations in arousal and reactivity), the depression score, age, and gender in the analysis.

Finally, we compared the attitudes towards mental health services as measured by IASMHS to those reported by other studies with different samples.

## 3. Results

The descriptive statistics of IASMHS scales are presented in Table 5. The mean sum score of the CTQ-SF values was 69.17 (*SD* = 12.02). This was significantly higher than the mean sum scores of a representative adult sample of the German population (*M* = 35.99, *SD* = 10.48; *t*(2718) = 44.46, *p* < .001; Wingenfeld et al., 2010) and of a large German sample of adult psychiatric patients (*M* = 48.3, *SD* = 19.6; *t*(1742) = 15.38, *p *< .001; Iffland, Brähler, Neuner, Häuser, & Glaesmer, 2013). The participants experienced a mean of 5.65 life events (*SD* = 3.09) during adulthood according to the LEC-5. This was similar to the average number of potentially traumatic lifetime events in a sample of patients with a substance use disorder and high rates of child abuse (*M* = 5.22, *SD *= 5.06; *t*(311) = 0.92, *p* = .359; Weiss, Tull, Lavender, & Gratz, 2013) and a sample of individuals who were referred to psychological therapy in a trauma centre (*M* = 5.00, *SD *= 2.48; *t*(411) = 2.34, *p* = .650; Karatzias et al., 2016).

**Table 1.** CFA and bifactor model fit indices for IASMHS.

	χ^2^	*df*	CFI	TLI	RMSEA (90% CI)	BIC^a^
One-factor model	931.1*	252	.705	.677	.111 (.104;.119)	17182.95
Three-factor model	438.6*	249	.918	.909	.059 (.050;.068)	16768.05
Bifactor model	395.5*	228	.927	.912	.058 (.048;.068)	16831.07

^a^Estimated using robust maximum likelihood estimation (MLR); *significant relative to degrees of freedom, *p* < .01.

**Table 2.** Standardized and unstandardized factor loadings including standard errors and interfactor correlations for the three-factor model of IASMHS.

Scale	(#) Item	β	B	SE
PO	(1) ‘There are certain problems which should not be discussed outside of one’s immediate family’	0.30	1.00	-
	(4) ‘Keeping one’s mind on a job is a good solution for avoiding personal worries and concerns’	0.23	0.78	0.31
	(7) ‘It is probably best not to know everything about oneself’	0.33	1.13	0.41
	(9) ‘People should work out their own problems; getting professional help should be a last resort’	0.79	2.67	0.76
	(12) ‘Psychological problems, like many things, tend to work out by themselves’	0.65	2.19	0.66
	(14) ‘There are experiences in my life I would not discuss with anyone’	0.48	1.62	0.46
	(18) ‘There is something admirable in the attitude of people who are willing to cope with their conflicts and fears without resorting to professional help’	0.56	1.89	0.57
	(21) ‘People with strong characters can get over psychological problems by themselves and would have little need for professional help’	0.55	1.86	0.56
HSP	(2) ‘I would have a very good idea of what to do and who to talk to if I decided to seek professional help for psychological problems’	0.50	0.66	0.09
	(5) ‘If good friends asked my advice about a psychological problem, I might recommend that they see a professional’	0.60	0.78	0.08
	(8) ‘If I were experiencing a serious psychological problem at this point in my life, I would be confident that I could find relief in psychotherapy’	0.82	1.07	0.06
	(10) ‘If I were to experience psychological problems, I could get professional help if I wanted to’	0.52	0.68	0.10
	(13) ‘It would be relatively easy for me to find the time to see a professional for psychological problems’	0.63	0.82	0.07
	(15) ‘I would want to get professional help if I were worried or upset for a long period of time’	0.88	1.14	0.07
	(19) ‘If I believed I was having a mental breakdown, my first inclination would be to get professional attention’	0.77	1.00	-
	(22) ‘I would willingly confide intimate matters to an appropriate person if I thought it might help me or a member of my family’	0.76	0.99	0.07
IS	(3) ‘I would not want my significant other (spouse, partner, etc.) to know if I were suffering from psychological problems’	0.74	1.14	0.11
	(6) ‘Having been mentally ill carries with it a burden of shame’	0.64	1.00	-
	(11) ‘Important people in my life would think less of me if they were to find out that I was experiencing psychological problems’	0.71	1.11	0.09
	(16) ‘I would be uncomfortable seeking professional help for psychological problems because people in my social or business circles might find out about it’	0.86	1.34	0.12
	(17) ‘Having been diagnosed with a mental disorder is a blot on a person’s life’	0.62	0.97	0.10
	(20) ‘I would feel uneasy going to a professional because of what some people would think’	0.86	1.34	0.13
	(23) ‘Had I received treatment for psychological problems, I would not feel that it ought to be “covered up”’	0.25	0.38	0.17
	(24) ‘I would be embarrassed if my neighbour saw me going into the office of a professional who deals with psychological problems’	0.77	1.19	0.12
	PO	IS		
IS	.52	-		
HSP	.70	.25		

PO = Psychological openness, IS = Indifference to stigma, HSP = Help seeking propensity; all but one factor loadings were statistically significant at *p* < .01; item 23 was significant at *p *< .05

**Table 3.** Binominal logistic regression analysis to predict current MHS use.

Variable	B	SE	OR (95% CI)
PO	0.03	0.03	1.03 (0.97;1.10)
HSP	0.09*	0.03	1.10 (1.03;1.17)
IS	−0.04	0.03	0.96 (0.91;1.01)
PTSD intrusion	0.52*	0.19	1.68 (1.15;2.45)
PTSD avoidance	−0.13	0.16	0.88 (0.65;1.19)
PTSD negative alterations in cognition and mood	0.57	0.32	1.76 (0.94;3.29)
PTSD alterations in arousal and reactivity	0.33	0.29	1.39 (0.79;2.43)
Depression	−0.63*	0.26	0.53 (0.32;0.90)
Age	−0.04	0.02	0.96 (0.93;1.00)
Gender (male)	−0.51	0.34	0.60 (0.31;1.16)

PO = Psychological openness, IS = Indifference to stigma, HSP = Help seeking propensity, PTSD = Posttraumatic stress disorder; *p < .05

**Table 4.** Bivariate correlations of variables included in regression model.

	2	3	4	5	6	7	8	9	10
1 PO	.414**	.377**	−.076	−.090	−.185**	−.170*	−.125	−.087	.123
2 HSP		.149*	.041	.163*	.150*	.094	.047	−.108	.162*
3 IS			−.290**	−.262**	−.170*	−.298**	−.193**	.093	.095
4 Depression				.574**	.405**	.769**	.660**	−.284**	−.055
5 PTSD intrusion					.577**	.663**	.625**	−.304**	.076
6 PTSD avoidance						.501**	.421**	−.203**	.135*
7 PTSD negative alterations^a^							.755**	−.396**	.022
8 PTSD alterations in arousal^b^								−.338**	.054
9 Age									−.135*
10 Gender									

Pearson correlation coefficients of variables included in the regression model; point biserial correlation coefficient for gender; ^a^ negative alterations in cognition and mood; ^b^ alterations in arousal and reactivity; *p < .05; **p < .01.

**Table 5.** Characteristics of study population and comparison with selected IASMHS samples.

Scale	Study Sample			^a^Watson & Hunter			^b^Drapeau, Cerel, & Moore			^c^Mesidor & Sly			^d^Troeung, Gasson, & Egan		
	*M*	*SD*	95% CI	*M*	*SD*	*t*(*df*)^e^	*M*	*SD*	*t*(*df*)	*M*	*SD*	*t*(*df*)	*M*	*SD*	*t*(*df*)
PO	17.33	6.48	[16.46, 18.19]	15.66	5.41	2.20 (313)*	-	-	-	17.01	5.60	0.44 (329)	15.61	8.82	2.48(545)*
HSP	25.03	6.01	[25.03, 26.63]	18.57	5.60	8.93 (313)***	25.97	5.14	2.07 (636)*	21.52	5.88	5.05 (329)***	24.10	6.69	1.66 (545)
IS	24.16	6.85	[23.24, 25.07]	16.83	9.18	7.83 (313)***	24.50	5.94	0.65 (636)	20.22	5.00	5.38 (329)***	-	-	-
Full scale	67.32	14.25	[65.41, 69.22]	-	-	-	74.76	12.80	6.71 (636)***	57.92	11.54	6.02 (329)***	-	-	-

PO = Psychological openness, HSP = Help seeking propensity, IS = Indifference to stigma; ^a^Watson and Hunter (2015): *N* = 95, African American women with possible anxiety/depression symptoms;^b^ Drapeau et al. (2016): *N *= 418, suicide-bereaved adults; PO subscale was excluded due to low internal consistency (α = 0.68);^c^ Mesidor and Sly (2014): *N *= 111, African American College Students with possible psychological distress;^d^ Troeung et al. (2015): *N *= 327, Australian adults with Parkinson’s Disease and possible mental health problems;^e^ *t*-tests comparing study sample to previous research samples; *p < .05; **p < .01; ***p < 0.001.

The mean scores for the PCL-5 subscales were: intrusion: 1.59 (*SD* = 1.27), avoidance: 1.59 (*SD* = 1.36), negative alterations in cognition or mood: 1.18 (*SD* = .97), arousal and reactivity: 1.49 (*SD* = .91). The mean score for the BSI depression scale was 1.02 (*SD* = 1.02). In our sample, 94 individuals (42.7%) were currently using mental health services.

The fit indices of the factor structure analyses of IASMHS are presented in Table 1. The one-factor model did not provide good fit to the data according to all fit indices and also had the highest BIC. The three-factor model and the bifactor model had similar fit, however the three-factor model clearly had the lower BIC and was thus superior to the bifactor model. Therefore, in our sample, the proposed three-factor structure of IASMHS provided the best fit to the data and was retained for further analyses. Table 2 shows the factor loadings of all items in the three-factor model. Several items of the psychological openness scale loaded only weakly on their according factor (β < .50): item 1, item 4, item 7, and item 14. One item of the indifference to stigma scale loaded weakly on its factor (item 23). The inter-factor correlations varied from *r* = .25 to *r* = .70.

We conducted a binomial logistic regression analysis to predict current mental health service use (Table 3). All bivariate correlations of the included variables are displayed in Table 4. The regression model correctly predicted 73.9% of the cases (78.4% of the no current mental health service use group and 67.7% of the current mental health service use group; *χ* ^2^ (*df* = 10) = 68.0, *p* < .001) and explained 36% of the variance (Nagelkerkes R^2^ = .36, Cox & Snell R^2^ = .27). The Wald statistic demonstrated that the help-seeking propensity scale, the PTSD-intrusion scale, and the depression scale made a significant contribution to the prediction. To rule out a possible bias due to those IASMHS items that loaded only weakly on their according factors, we repeated the regression analysis with a reduced set of items based on the results of the CFA: items 1, 4, 7, and 14 were omitted from the psychological openness scale and item 23 was omitted from the indifference to stigma scale. The results of the regression analysis with those reduced scales did not differ from those with the full scales and are therefore not presented here in detail.

Table 5 compares the results obtained from IASMHS scale in the present study with results of previous research. The current sample was characterized by significantly higher levels in IASMHS scales compared to most of these distressed samples (Mesidor & Sly, 2014; Troeung et al., 2015; Watson & Hunter, 2015). However, a sample of suicide-bereaved adults reported higher levels in the help-seeking propensity and the full IASMHS scale (Drapeau et al., 2016).

## 4. Discussion

In the present study we aimed to investigate the psychometric properties of IASHMS, to ascertain the predictive power of IASMHS beyond specific variables, and to compare help-seeking attitudes between institutional abuse survivors and other samples with possible mental health problems.

We replicated the proposed factor structure of IASMHS (Mackenzie et al., 2004) in the institutional abuse sample. Although a three-factor bifactor model fit the data well, the confirmatory factor analysis supported the three-factor model and confirmed the intended three-factor theory of IASMHS (Hyland et al., 2014; Mackenzie et al., 2004). However, some items (1, 4, 7, 14) of the psychological openness scale loaded weakly on their according factor, which corresponded with the findings of Hyland et al. (2014). It might be reasonable to adjust these items in future research with the goal to better represent the characteristics of this subscale. For example, item 4 ‘Keeping one’s mind on a job is a good solution for avoiding personal worries and concerns’ could also be understood as a healthy coping strategy of individuals that acknowledge their psychological problems and seek professional help. The items of the indifference to stigma and help-seeking propensity scale loaded adequately on their corresponding factors. The internal consistency of IASHMS was – with the exception of the psychological openness scale – similar to the results of prior research (Mackenzie et al., 2004).

To assess the predictive validity of IASMHS as part of a criterion-oriented validation process (Cronbach & Meehl, 1955), we investigated the predictive power of the three IASMHS scales for current mental health service use, when also including PTSD, depression, age, and gender as predictors in the model. The results showed that higher scores in the help-seeking propensity and the PTSD-intrusion scales, and lower scores in the depression scale contributed significantly to the prediction of current mental health service use. The help-seeking propensity scale refers to an individuals’ intention and ability to seek help. Similarly, Mackenzie et al. (2006) found a strong predictive association between help-seeking propensity and intentions to seek professional help. Intentions in turn were described as the strongest predictor for actual help-seeking behaviour (Ajzen, 1985). Help-seeking propensity emerged as a reliable predictor of intentions to mental health service use in several studies (e.g. Hyland et al., 2014; Mackenzie et al., 2004; Mesidor & Sly, 2014; Troeung et al., 2015). Single studies indicated significant – although weaker – correlations between mental health service use and psychological openness (Hyland et al., 2014), or indifference to stigma (Mackenzie et al., 2004). In one case the psychological openness scale was removed due to low internal consistency (Drapeau et al., 2016). We suppose that sample characteristics contributed to these differences in at least two possible ways. First, we assume that the relationship between psychological openness and mental health service use might be affected by sample-specific aspects such as the experience of institutional betrayal (Smith & Freyd, 2014). Survivors of institutional abuse experienced violent abuse of power during their youth by authorities whose duty was to protect and care for them. Those experiences might result in suspiciousness towards authorities, such as mental health professionals. This might be associated with a complex interaction between psychological openness and mental health service use. Second, all participants were offered financial compensation and psychotherapy by the victim protection commission, although not all participants underwent treatment. It is possible that this lowered the predictive power of both the psychological openness and stigma scales compared to other samples. Furthermore, it should be considered that these comparisons represent a first exploratory approach towards the integration of existing research on mental health service use and attitudes in distressed samples. This comparison might be biased by the heterogeneity of the samples and should be investigated systematically in future research.

We found, in a systematic review on perceived barriers and facilitators to mental health service use, that PTSD-symptoms, according to re-experiencing, prevent trauma survivors from engaging in professional treatment (Kantor, Knefel, & Lueger-Schuster, 2017); intrusion predicted mental health service use in the present study. A possible explanation may be that severe psychopathology leads to higher psychological strain and therefore a wish for change (Bicanic, Snetselaar, de Jongh, & van de Putte, 2014; Gavrilovic et al., 2005; Kantor et al., 2017). In contrast, depression deterred participants from help-seeking which could be attributed to the nature of depression including fatigue, loss of activities, and hopelessness (Sherwood, Salkovskis, & Rimes, 2007).

Studies investigating attitudes towards mental health service use in samples with possible mental health problems found homogenous profiles in the relative differences of IASMHS scales. The help-seeking propensity scale frequently showed higher levels than the psychological openness scale, whereas the indifference to stigma subscale showed the lowest levels among the scales. However, the absolute scale-levels differed significantly between samples: African Americans and adults with Parkinson’s Disease showed lower scores in IASMHS scales (Mesidor & Sly, 2014; Troeung et al., 2015; Watson & Hunter, 2015). Only suicide bereaved adults reported significantly higher scores in two of the three scales compared to the institutional abuse sample (Drapeau et al., 2016). Compared to other samples, institutional abuse survivors showed relatively positive attitudes which could be explained by their psychotherapy experience (Gavrilovic et al., 2005) and lower barriers to service use in the course of the governmental recompense process. Future research should seek to determine the change in attitudes in relation to prior psychotherapy experiences, as well as the longitudinal development of attitudes regarding symptom severity and trauma experiences. Practical implementations should target help-seeking propensity in order to facilitate intention (psycho-education) and perceived ability to seek help (lowering external barriers). Online tools and mobile applications, for instance, have the potential to contribute to help-seeking propensity as they are increasingly available, are barrier-free, have a wide reach, and offer psycho-education and useful therapeutic interventions (Olff, 2015; Ruzek et al., 2011).

This is the first study investigating the psychometric properties of IASMHS in a trauma sample that was screened for traumatic experiences and PTSD-symptoms. Further, in contrast to earlier studies we examined the current mental health service use rather than intentions to use. However, there are a number of limitations that should be noted. First, although institutional abuse is a worldwide phenomenon and our results may thus generalize to other adult survivors of abuse in institutional settings, generalizability may still be limited by the use of cross-sectional data and this specific sample of trauma survivors. Second, although we encouraged the participants to answer openly, as with all research on attitudes the participants’ answers might be biased towards social desirability. Third, prior positive psychotherapy experiences – even decades ago – might have influenced participants current attitudes, which was not assessed in this study and can thus not be ruled out. Fourth, in order to differ mental health service use from non-use we defined treatment-use as receiving a minimum of eight therapy sessions within 12 months. We applied this definition for the purpose of establishing comparability across studies using the same cut-off value (Wang et al., 2000). However, using a different cut-off value might be associated with different results. Fifth, a quantitative assessment might be restricted in its potential to map the complex process underlying mental health service use and could be enriched by a qualitative approach.

## 5. Conclusion

Understanding institutional abuse survivors’ attitudes towards mental health service use seems to be crucial to facilitate treatment use. IASMHS is a useful instrument in assessing survivors’ attitudes, even if some items might benefit from an adjustment. Post-trauma interventions should target trauma survivors’ help-seeking propensity to facilitate mental health service use.
